# Are you Ernest Shackleton*,* the polar explorer? Refining the criteria for delirium and brain dysfunction in sepsis

**DOI:** 10.1186/s40560-017-0218-z

**Published:** 2017-03-07

**Authors:** Frank Anthony Rasulo, Giuseppe Bellelli, Eugene Wesley Ely, Alessandro Morandi, Pratik Pandharipande, Nicola Latronico

**Affiliations:** 1 Department of Anesthesiology, Critical Care Medicine and Emergency, Division of Neurocritical Care, Spedali Civili University Hospital, Piazzale Ospedali Civili, 1, 25123 Brescia, Italy; 2 Department of Medical and Surgical Specialties, Radiological Sciences and Public Health, University of Brescia, Brescia, Italy; 3 Geriatric Research Group, Brescia, Italy; 4 School of Medicine and Surgery, University of Milano-Bicocca, Milan, Italy; 5 Geriatric Unit, San Gerardo Hospital, Monza, Italy; 6 Center for Quality Aging, Vanderbilt University School of Medicine, Nashville, TN USA; 7 Division of Allergy, Pulmonary, and Critical Care Medicine, Vanderbilt University School of Medicine, Nashville, TN USA; 8 Center for Health Services Research, Vanderbilt University School of Medicine, Nashville, TN USA; 9 Geriatric Research, Education, and Clinical Center, Department of Veterans Affairs Medical Center, Tennessee Valley Health Care Center, Nashville, TN USA; 10 Department of Rehabilitation and Aged Care of the Fondazione Camplani, Ancelle Hospital, Cremona, Italy; 11 Department of Anesthesiology, Division of Critical Care, Vanderbilt University School of Medicine, Nashville, USA; 12 Anesthesia Service, Department of Veterans Affairs, Tennessee Valley Healthcare System, Nashville, USA; 13 Department of Anesthesiology, Critical Care Medicine and Emergency, Spedali Civili University Hospital, Brescia, Italy

**Keywords:** Third International Consensus Definitions for Sepsis and Septic Shock, Sepsis, Quick Organ Failure Assessment (qSOFA) score, Glasgow Coma Scale (GCS) score, Delirium, Brain dysfunction

## Abstract

The Third International Consensus Definitions for Sepsis and Septic Shock has recently defined sepsis as a life-threatening organ dysfunction caused by a dysregulated host response to infection. Organ dysfunctions in this consensus definition were identified as an organ-specific Sequential [Sepsis-related] Organ Failure Assessment (SOFA) score ≥ 2 points. The quick SOFA (qSOFA) considers altered mentation indicating brain dysfunction when the Glasgow Coma Scale (GCS) score is ≤13 or ≤14. However, concern has been expressed that the revised criteria may lead to a failure in recognizing the signs of potentially lethal organ dysfunction and thus sepsis. Patients with delirium have a fluctuating course, and GCS can be normal or only slightly reduced at the time when signs of delirium are already present. We here report an illustrative case showing how an acute, initially unrecognized, urinary tract infection caused acute brain dysfunction with profound behavioral and cognitive dysfunction despite normal GCS, hence not meeting the criteria for sepsis.

## Background

The Third International Consensus Definitions for Sepsis and Septic Shock has recently defined sepsis as a life-threatening organ dysfunction caused by a dysregulated host response to infection [1]. Organ dysfunction is defined as an organ-specific Sequential [Sepsis-related] Organ Failure Assessment (SOFA) score ≥ 2 points. Outside of the ICU, where all components of SOFA are not routinely measured, the quick SOFA (qSOFA) score has been proposed as a surrogate for SOFA. Within the qSOFA, the Glasgow Coma Scale (GCS) is used to evaluate neurological dysfunction; however, the cut-off score remains uncertain. In fact, the predictive validity of qSOFA is not significantly different when using a GCS score of <15 compared with GCS score of ≤13. Moreover, delirium may be present before other signs and symptoms indicative of infection and sepsis appear, but it is not considered in the new criteria for defining sepsis. Sepsis and septic shock are both associated with an increase in mortality in hospitalized patients. It is, therefore, paramount to diagnose brain dysfunction as soon as possible in order to promptly initiate treatment.

We here report an illustrative case showing how an acute, initially unrecognized urinary tract infection caused acute brain dysfunction with profound behavioral and cognitive dysfunction despite normal GCS, hence not meeting the criteria for sepsis.

## Main text

In the summer of 2014, an 86-year-old man returned from the wine cellar with a bottle of wine to accompany his meal, but was found to be wearing his parka, hood, and gloves. Thinking it was a prank, his son asked him, “Hello, Sir Shackleton”, referring to the famous polar explorer Sir Ernest Henry Shackleton, “is anyone else from your crew joining us for dinner?” However, the son, a Doctor, quickly realized that something was wrong when his father was not amused by the joke. The elderly gentleman did finally remove the excess clothing and sat back down to finish his meal, though appeared intermittently confused.

Later in the evening, he continued presenting fluctuating signs of confusion with difficulty focusing attention and keeping track of what was being said and had episodes of incoherent thinking. He was unable to tell the months backwards and was continuously distracted by home stimuli. Responses to simple questions such as “does a stone float on water” were inappropriate. No signs of altered consciousness were present; GCS remained most of the time at 15 (normal score), but sometimes fluctuated to 14 (confusion at verbal assessment). This fluctuation in mental status accompanied by inattention prompted his son in performing a more thorough examination which revealed heavy breathing, frequent and painful urination, and slight fevers, leading to a diagnosis of a urinary tract infection. Antibiotics (sulfamethoxazole and trimethoprim) were started, and 2 days later the patient returned to his baseline neurological status but was unaware of the behavioral and cognitive deficits he had suffered in the previous days.

According to the new criteria for defining sepsis, either a GCS of less than 15 or GCS ≤ 13, can be used to define neurologic dysfunction with qSOFA [1], which makes different series difficult to compare. In fact, three recent papers used the two different GCS cut-off scores, two of which used <15 [2, 3] and one ≤13 [4]. Work is still needed to determine the optimal way to identify patients with sepsis on the wards that will most likely benefit from earlier and more aggressive interventions [5].

The gentleman in our report manifested all signs and symptoms of delirium [6]. This acute brain failure following infection should not be ignored and shows that he had sepsis, which would portend a higher morbidity and mortality as compared to a simple infection, but would risk going unrecognized if routine delirium monitoring is not performed. Delirium prompted investigations into infection as the possible cause, thereby permitting timely antibiotic treatment and resolution of the clinical condition. Had we based our diagnosis on an altered mental status as defined by a GCS ≤ 13, this patient would have had a simple infection, but not sepsis. Delirium is common, associated with worsening of outcome if not recognized and is often the first manifestation of sepsis [7]. Early identification and management of sepsis in the wards or the ICU may also prevent worsening and development of septic shock and its associated outcomes. The prevalence of delirium ranges from 8 to 80%, with the highest prevalence in the ICU and after surgical procedures, and its occurrence is associated with increased risk of mortality, longer ICU and hospital stay, higher costs, and increased risk of developing dementia or worsening of a pre-existing dementia and functional decline [7, 8]. Given that delirium can be reliably diagnosed through use of standardized, validated, and easy to use tools, such as the Confusion Assessment Method (CAM), CAM-ICU, Intensive Care Delirium Screening Checklist, and 4AT, there appears to be scant reason to not incorporate it into the identification algorithms for brain organ dysfunction accompanying infection [9].

## Conclusions

Unexplained organ dysfunctions should raise the possibility of an underlying infection being the provocateur, and it is unclear what lower limit of GCS should be used to uncover brain dysfunction in sepsis. Any new conference to re-evaluate criteria for sepsis, with a broader base of constituents, should also consider delirium to refine the criteria for sepsis.

## Abbreviations

4AT: Assessment test for delirium and cognitive impairment
CAM: Confusion Assessment Method
CAM-ICU: Confusion Assessment Method for the Intensive Care Unit
GCS: Glasgow Coma Scale
ICU: Intensive care unit
qSOFA: quick Sequential Organ Failure Assessment
SOFA: Sequential Organ Failure Assessment
